# Investigation of a Cluster of Neural Tube Defects — Central Washington, 2010–2013

**Published:** 2013-09-06

During August 2012, a health-care provider in central Washington alerted the Washington State Department of Health (DOH) about an excessive number of anencephaly births at a local hospital. After examining referral patterns for high-risk pregnancies in central Washington, DOH identified pregnancies affected by a severe neural tube defect (NTD) in a three-county area. Case findings included a review of area hospital discharge records for *International Classification of Diseases, Ninth Revision* codes 740, 741, 742, or 655.0; vital statistics reports; and perinatology office records. From these sources, 27 confirmed NTD-affected pregnancies occurring during January 2010–January 2013 were identified among women residing in the three-county area. Twenty-three pregnancies were affected by anencephaly, three with spina bifida, and one with encephalocele. The anencephaly rate was 8.4 per 10,000 live births (95% confidence interval [CI] = 4.5–12.0), compared with a national estimate of 2.1 per 10,000 live births (CI = 1.9–2.2) (1). In contrast, the rate of spina bifida was 1.3 per 10,000 live births (CI = 0.3–3.8), compared with 3.5 per 10,000 live births nationally (CI = 3.3–3.7) (1).

During February 2013, a case-control study was conducted by abstracting prenatal records from the 27 NTD-affected pregnancies and 108 randomly selected control subject pregnancies in women who had received care at the same 13 prenatal clinics. Control subjects were matched to case-patients by the month and year of last menstrual period. Eligibility criteria for control subjects included a pregnancy without an indication of a structural or genetic birth defect during routine prenatal care and prenatal residence in one of the three study counties. Information abstracted from medical records included sociodemographic characteristics, maternal and paternal occupations, maternal smoking and alcohol use, pregnancy health conditions (e.g., anemia, diabetes, or infectious diseases), parity, gravidity, prepregnancy height and weight, and medication use (including over-the-counter remedies, vitamins, and folic acid supplementation). Residential address during pregnancy was used to determine use of public versus private well-water supply.

No statistically significant differences were identified between cases and controls, and a clear cause of the elevated prevalence of anencephaly was not determined. DOH recommended reminding doctors about the importance of folic acid supplementation for women of childbearing age (2), and monitoring private well nitrate concentrations because of their potential association with birth defects and other adverse health outcomes (3). Active surveillance of new NTD cases began February 2013 and will continue through 2013.

## Reported by

*Amy Person, MD, Benton-Franklin Health District; Chris Spitters, MD, Yakima Health District; Glen Patrick, MPH, Cathy Wasserman, PhD, Patrick Vander Kelen, PhD, Juliet VanEenwyk, PhD, Washington State Dept of Health. Suzanne Gilboa, PhD, James Kucik, PhD, Div of Birth Defects and Developmental Disabilities, National Center on Birth Defects and Developmental Disabilities; Reed Sorenson, MPH, Council of State and Territorial Epidemiologists Fellow; Elizabeth Ailes, PhD, Mandy Stahre, PhD, EIS officers, CDC.* ***Corresponding contributor:*** *Mandy Stahre, mstahre@cdc.gov, 360-236-4247.*
